# The Contribution of Non-Professional Antigen-Presenting Cells to Immunity and Tolerance in the Liver

**DOI:** 10.3389/fimmu.2018.00635

**Published:** 2018-03-28

**Authors:** Christina Mehrfeld, Steven Zenner, Miroslaw Kornek, Veronika Lukacs-Kornek

**Affiliations:** Department of Medicine II, Saarland University Medical Center, Homburg, Germany

**Keywords:** antigen presentation, liver, tolerance, immunoregulation, CD1d

## Abstract

The liver represents a unique organ biased toward a tolerogenic milieu. Due to its anatomical location, it is constantly exposed to microbial and food-derived antigens from the gut and thus equipped with a complex cellular network that ensures dampening T-cell responses. Within this cellular network, parenchymal cells (hepatocytes), non-parenchymal cells (liver sinusoidal endothelial cells and hepatic stellate cells), and immune cells contribute directly or indirectly to this process. Despite this refractory bias, the liver is capable of mounting efficient T-cell responses. How the various antigen-presenting cell (APC) populations contribute to this process and how they handle danger signals determine the outcome of the generated immune responses. Importantly, liver mounted responses convey consequences not only for the local but also to systemic immunity. Here, we discuss various aspects of antigen presentation and its consequences by the non-professional APCs in the liver microenvironment.

## Introduction

The liver is the metabolic center of the body that is critical for maintaining homeostasis. Additionally, it perpetuates a tolerogenic environment, is involved in peripheral tolerance, e.g., against food-derived antigens, and carries various immunological functions affecting not only the local but also the systemic immunity (1–3). To fulfill such diverse roles, the liver is provided with blood by the hepatic artery as well as by the portal vein (4). The portal vein not only carries nutrient rich blood from the gut but it also contains molecules/antigens derived from the digested food and the gut microbiome (5). In such exposed microenvironment, it is critical how the liver handles antigens and danger signals and how it allows the maintenance of the tolerogenic milieu while staying alert and ensuring the generation of liver-protecting T-cell responses.

**Figure 1:**
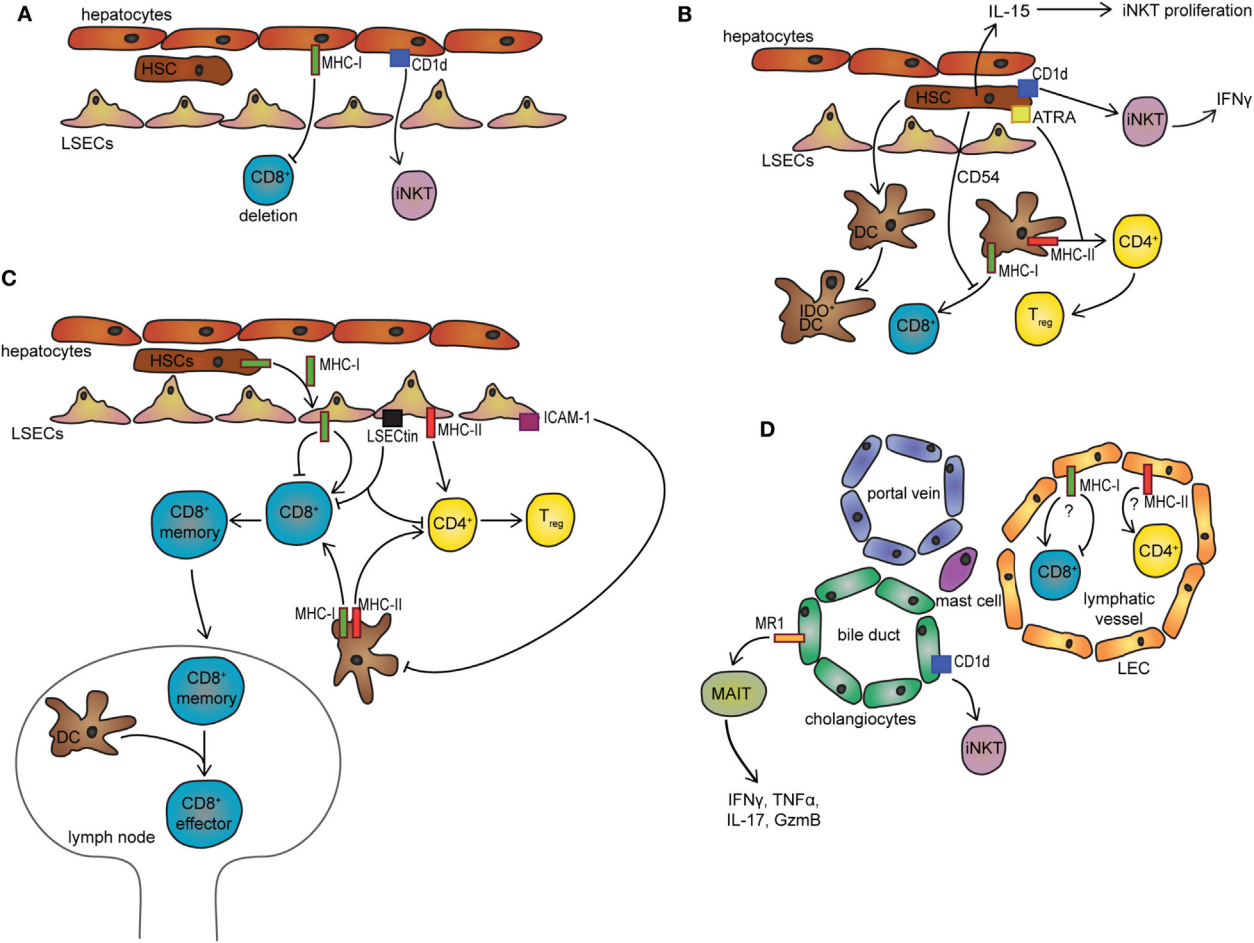
Non-professional APCs in the liver microenvironment. **(A)** Presentation of exogenous antigen to CD8^+^ T-cells by hepatocytes leads to T-cell deletion. *Via* CD1d, hepatocytes can activate iNKT cells. **(B)** HSCs inhibit DC-mediated activation of CD8^+^ T-cells *via* CD54 and promote DC-mediated differentiation of CD4^+^ T-cells to T_regs_ using all-trans retinoid acid. HSCs induce IDO expression in DCs upon direct contact. Additionally, *via* CD1d HSCs can induce IFNγ secretion in iNKT cells and promote their proliferation by providing IL-15. **(C)** LSECs promote the differentiation of CD4^+^ T_regs_ or CD8^+^ memory T-cells, respectively. CD8^+^ memory T-cells migrate to the lymph nodes where they can be reactivated by DCs. LSECs can inhibit DC-mediated antigen presentation *via* ICAM1 and inhibit T-cell activation *via* LSECtin. LSECs receive MHC-I antigen complexes from HSCs *via* transcytosis. **(D)** In the portal triad, cholangiocytes can activate MAIT cells *via* MR1 and iNKT cells *via* CD1d. Additionally, LECs and mast cells could represent a potential cell population with MHC-I and MHC-II antigen-presenting ability. LSECs, liver sinusoidal cells; HSC, hepatic stellate cell; ATRA, all-trans retinoid acid; LEC, lymphatic endothelial cell; MAIT, mucosal-associated invariant T-cell; DC, dendritic cell; IDO, indoleamine 2,3-dioxygenase; IL, interleukin; IFN, interferon; iNKT, invariant natural killer cell; T_regs_, regulatory T-cells; MHC, major histocompatibility complex; MR1, MHC class I-like-related molecule; APCs, antigen-presenting cells; LSECs, liver sinusoidal endothelial cells.

The liver consists of parenchymal cells (such as hepatocytes and cholangiocytes), liver sinusoidal endothelial cells (LSECs), hepatic stellate cells (HSCs), and a complex immune cell network built by myeloid and lymphoid cell populations (Figure 1). Sinusoids are lined by LSECs and provide docking sites for immune cells (6). HSCs in the space of Dissé can regulate the blood flow (7) and represent the main reservoir of vitamin A (8, 9). Hepatocytes carry out complex metabolic functions and secrete the bile on their basolateral surface into the bile canaliculi (10). Nutrients and molecules from the blood can reach hepatocytes *via* the fenestrated layer of LSECs containing oval pores approximately 50–150 nm in diameter (11, 12). Additionally, LSECs are able to trancytose blood-derived materials directly to hepatocytes (12, 13). To assure that hepatocytes can perform their metabolic function, the liver receives nearly 25% of the cardiac output (4). Besides its large blood flow, the liver produces between 25 and 50% of the total lymph arriving in the thoracic duct (14, 15). Lymphatic endothelial cells (LECs) lining the lymphatics can be mainly found in the portal area (15) and provide important transport route for immune cells such as dendritic cells (DCs) and memory T-cells (16) (Figure 1D).

Most antigens in the liver are taken up and processed by professional antigen-presenting cells (APCs) such as DCs, Kupffer cells (KCs), or monocyte-derived myeloid cells (17). These cells are important milestones in generating liver-protective immunity as well as tolerance and have been recently discussed elsewhere (17, 18). In this review, we will summarize the antigen presentation and its consequences by non-professional APCs in the liver.

## Presentation of Antigens on Major Histocompatibility Complex (MHC) Molecules

### Liver Sinusoidal Endothelial Cells

Due to the direct contact with blood and its carried substances, it is not surprising that LSECs possess very efficient endocytic capacity that is superior to any professional APCs within the body (19, 20). To fulfill their engulfing potential, LSECs express various scavenger receptors (e.g., Stabilin 1, 2, and B1), lipoprotein receptor-related protein-1, and a range of C-type lectin receptors (21–23). LSECs efficiently endocytose soluble molecules or particles under 200 nm, whereas KCs attached to LSECs within the sinusoids cover particles and debris exceeding 200 nm (24). Together, they create a well-controlled functional dichotomy for constantly probing the liver environment.

Liver sinusoidal endothelial cells constitutively carry low level of MHC-II and are able to upregulate its expression upon exposure to inflammatory cytokines (25, 26). Naïve CD4^+^ T-cells primed by LSECs under steady state differentiate into regulatory T-cells (T_regs_) that lack the transcription factor Forkhead-Box-Protein P3 (FoxP3), which is normally expressed by T_regs_ generated by professional APCs (27). These LSEC-induced CD25^low^FoxP3^−^ T-cells are very immune suppressive (27). This aspect of LSEC-mediated antigen presentation could provide therapeutic benefits. Nanoparticles loaded with autoantigens are taken up by LSECs and lead to MHC-II presentation and to the consequent induction of regulatory CD4^+^ T-cells (28). Importantly, LSEC-targeted nanoparticles were able to reverse experimental autoimmune encephalomyelitis *in vivo* (28).

Liver sinusoidal endothelial cells are not only able to present exogenous antigen on MHC-II but also on MHC-I and thus capable of cross-presentation (Figure 1C) (29). Surprisingly, LSECs can cross-present soluble antigens even more efficiently than DCs (20, 30). This antigen presentation, however, is only limited to a short time period due to the efficient transcytotic transport (30). Besides soluble molecules, LSECs cross-present antigens from virus-infected hepatocytes (31), as well as cancer-associated antigens from apoptotic tumor cells (32). After encountering CD8^+^ T-cells, LSECs upregulate the co-inhibitory molecule B7-H1 (10-fold), therefore, shifting the balance from activation to tolerance induction in CD8^+^ T-cells (33). CD8^+^ T-cells primed by LSECs exhibit a phenotype (CD25^low^CD62L^high^) that is different from CD8^+^ T-cells activated by DCs (CD25^high^CD62L^low^) (33) and demonstrate a rapid yet transient induction of effector functions (33). Trans-signaling of IL-6 between LSECs and CD8^+^ T-cells during priming is responsible for the fast (only 18 h) activation and the expression of granzyme B (GzmB) (34). This IL-6 trans-signaling is necessary to make CD8^+^ T-cells susceptible toward IL-2 (35). Despite the effector capacity, LSEC-primed CD8^+^ T-cells remain refractory and at later time point are non-responsive to restimulation *via* the T-cell receptor (TCR) (33). Moreover, these T-cells show memory-like phenotype and are capable of migrating to lymphoid organs (e.g., lymph nodes) where they can support anti-infectious immunity upon simultaneous restimulation through the TCR and costimulatory molecules (36). This is highly relevant since the LSEC-primed quiescent CD8^+^ T-cells are not lost from the immunological T-cell repertoire and could be utilized upon infectious danger (36).

However, if antigen concentration is high *via* strengthening the MHC-TCR interaction, LSECs could mount efficient effector CD8^+^ T-cell activation as well (37). In this case, the co-inhibitory signal of PD1-PD-L1 axis is overcome by IL-2 released from activated CD8^+^ T-cells that lead directly to the differentiation of cytotoxic T-cells following antigen cross-presentation (37). Such a scenario could be a protective instrument for the liver for example in hepatitis B virus (HBV) infection where viral antigen expression is sufficiently high (38). Cross-presentation capacity of LSECs and consequent anti-viral cytotoxic T-Lymphocyte could be further enhanced through a mechanism differing from cross-dressing, where HSCs transfer MHC-I molecules to the LSEC-presentation machinery (39).

Besides direct antigen presentation, LSECs also affect bystander T-cell activation in the liver. *Via* LSECtin they can inhibit T-cell activation, proliferation, and effector function and *via* ICAM-1 hinder DC-mediated antigen presentation further supporting the maintenance of the tolerogenic milieu in the liver (40, 41). It is important to note that LSECs are not a solely tolerance-promoting machinery. Due to toll-like receptors and damage-associated molecular patterns, LSECs can sense danger signals. Although under steady state they are refractory to low dose of LPS, they instantaneously respond to LPS concentration change and alert the protecting acute phase response of the liver (42). Thus, they not only represent a key APC population but also central sentinels within the liver microenvironment.

### Hepatocytes

Despite the fact that hepatocytes are not in direct contact with the sinusoidal blood flow (Figure 1A), lymphocytes scan the surface of hepatocytes *via* their protruding filopodia through the openings of the fenestrated endothelium (43, 44). While this is part of immune homeostasis it also can give an opportunity for hepatocytes to present antigen to T-cells. Indeed, hepatocytes are capable of priming naïve CD8^+^ T-cells directly and *via* cross-presentation (20, 45) but fail to provide the activated CD8^+^ T-cells with the necessary survival factor and therefore cause CD8^+^ T-cell deletion (45, 46). A recent study showed that the pathway of antigen processing and loading of MHC-I complex depends on a specific chaperone in the endoplasmatic reticulum–Golgi intermediate compartment called collectrin (47). This protein is not expressed in KCs or DCs and indicates a distinct mechanism of antigen processing for cross-presentation in hepatocytes (47).

Steady-state tolerogenic priming, however, could be contradictory in the case of hepatocyte-trophic viral invasion. In viral infection, virus-positive hepatocytes are eliminated by activated circulating CD8^+^ T-cells either directly recognizing antigen on hepatocytes or indirectly *via* LSEC-mediated cross-presentation of infected hepatocytes and consequent tumor necrosis factor (TNF) release (31). Efficient T-cell immunity and the clearance of virus-infected hepatocytes are inversely correlated with the number of infected cells (48). Accordingly, higher the number of infected hepatocytes, less efficient is the response due to CD8^+^ T-cell exhaustion (48, 49). Thus, efficient viral clearance most likely depends on a division of labor by multiple non-immune (hepatocytes and LSECs) and immune cells (DCs) in the liver microenvironment.

Hepatocytes do not express MHC-II molecules under steady-state condition (50); however, they could acquire during inflammation (50). MHC-II overexpressing hepatocytes were also capable of activating CD4^+^ T-cells *in vitro* (50). Accordingly, hepatocyte-specific expression of neural autoantigen led to the generation of CD4^+^CD25^+^FoxP3^+^ T_regs_, which protected against autoimmune encephalomyelitis (51). While the above-mentioned study assumed that antigen targeting to hepatocytes would equal to hepatocyte-mediated tolerance induction, the exact cell population that was responsible for the protective T_reg_ induction was not identified (51). Thus, cross-presentation of hepatocyte-derived material by an alternative liver cell population could not be excluded in this phenomenon. Nevertheless, this study raised an important point that hepatocyte targeting could be an alternative approach for autoimmune disease therapy.

In accordance with this, transgenic animals expressing ovalbumin in hepatocytes could mount OT-II cell activation and proliferation under steady state only in spleen and in draining hepatic lymph node and not within the liver (52). Moreover, this presentation depended on bone marrow-derived APCs (53) instead of hepatic immune or parenchymal cells (53). Thus, future studies are required to clarify hepatocyte-mediated CD4^+^ T-cell activation and its contribution to local and systemic immune responses.

### Hepatic Stellate Cells

The antigen-presenting capacity of HSCs is controversial. Although they express costimulatory molecules such as CD40, CD80 and some studies found that interferon (IFN)γ regulated MHC-II expression in HSCs (54–57), their antigen uptake capacity is rather low (20). This questions whether they would be efficiently able to function as APCs *in situ*. Nevertheless, murine HSCs demonstrated the ability to process and present exogenous soluble antigens and activate both naïve CD4^+^ and CD8^+^ T-cells *in vitro* and could generate efficient T-cell response upon adoptive transfer *in vivo* (Figure 1B) (55). In contrast to this, other studies show that HSCs are less effective in generating T-cell responses. They induce T-cell apoptosis through B7-H1 (58–60) and B7-H4 signaling (59). Importantly, these studies addressed T-cell responses generated by peptide-pulsed HSCs and thus eliminated the uptake and presentation process of the antigen. While the ability of antigen presentation of HSCs is in debate, considerable amount of studies confirmed their potent immunoregulatory capability. HSCs induce the generation of indoleamine 2,3-dioxygenase (IDO)^+^ DCs in a contact dependent-manner and trigger myeloid-derived suppressor cell differentiation upon exposure to monocytes (61, 62). Additionally, in the presence of DCs, HSCs direct naïve CD4^+^ T-cell activation toward T_reg_ differentiation (Figure 1B) (63), a process that is likely mediated by all-trans retinoid acid, a retinol metabolite (63). Moreover, HSCs display a veto function, because they inhibit the priming of CD8^+^ T-cells induced by DCs through a CD54-dependent mechanism (64). Thus, HSCs represent a key component in the tolerogenic liver milieu.

### Cholangiocytes

Cholangiocytes line the bile ducts and are exposed constantly to the bile content containing a wide variety of molecules. The ability of these cells to present antigen has been investigated in multiple studies. *In vivo* targeting of model antigen (ovalbumin) to cholangiocytes resulted in CD8^+^ T-cell activation in the liver and in the draining lymph node, but failed to induce CD4^+^ T-cell activation (52). This study has not identified the exact APC type in their transgenic system and thus antigen presentation and T-cell stimulatory capacity of cholangiocytes in the mouse liver remains elusive. Importantly, human cholangiocytes do not express costimulatory molecules (65) and are unable to induce CD4^+^ T-cell responses *in vitro* (66). Interestingly, cholangiocytes express human leukocyte antigen (HLA)-II molecules in human primary biliary cholangitis (PBC) (67), but the role in T-cell priming under pathogenic condition has not been investigated.

While MHC-I and MHC-II presentations of these cells are rather controversial, cholangiocytes can use an MHC-I-related molecule, MHC class I-like-related molecule (MR1) for inducing lymphocyte activation, which was shown in human liver (Figure 1D) (68). MR1 has the antigen-binding cleft for vitamin B metabolites derived from pathogenic/commensal bacteria (69) and is recognized by mucosal-associated invariant T-cells (MAITs) (70). In healthy and diseased liver, MAITs are abundant and mainly located around bile ducts and biliary epithelial cells (71). Activated MAIT cells are pro-inflammatory and secrete cytokines such as IFNγ, TNFα, IL-17, and GzmB (68). This mechanism is thought to protect the biliary tract from infiltrating commensal, as well as from pathogenic bacteria.

### Other Non-Professional APCs to Consider: LECs, Mast Cells, and Neutrophils

Lymphatic endothelial cells have a high endocytic capacity and within lymphoid organs are able to present exogenous antigen to T-cells on both MHC-I and MHC-II molecules (16, 72, 73). Additionally, they represent a stromal cell population expressing peripheral tissue restricted antigens and mediate the deletion of autoreactive CD8^+^ T-cells (16, 74, 75). Moreover, LECs are potent immunoregulators and can inhibit DC-mediated antigen presentation and bystander T-cell proliferation *via* direct contact and nitric oxide production (72, 76). Although higher abundance of LECs is associated with multiple liver disorders, their antigen presentation capacity to influence T-cell responses has not been investigated (Figure 1D). These aspects could be interesting to address in the future in the view of the tolerogenic liver environment.

Mast cells are primarily located around the portal triad and could be identified within lymphatic vessels (14, 77). Freshly isolated human mast cells do not express antigen-presenting molecules under steady state, but in the presence of IFNγ they upregulate HLA class II and costimulatory molecules (CD80 and CD40) (78). Consequently, they are able to activate CD4^+^ T-cells *in vitro* (78). The uptake of antigen is independent of IFNγ, and the antigen processing is associated with their secretory granules (78). Mast cells are more abundant in cholangiopathies such as primary sclerosing cholangitis (PSC) or PBC (77, 79), and mast cell-deficient mice are protected from liver damage induced by bile duct ligation (80). Whether their antigen-presenting capacity is relevant for these liver diseases remains to be elucidated.

Similar to mast cells, neutrophils are capable of functioning as APCs under inflammatory condition *in vitro* (81, 82). Because these cells are recruited in larger numbers in liver inflammation and especially during infection, their APC capacity might be considered in future studies influencing liver pathology.

## Presentation of Lipid Antigens on CD1D Molecule

The CD1 family of proteins is able to bind and present lipid antigens (83). Five members belong to this family, and they are divided into two groups. CD1a-c belong to the first group, which present various lipids from microbes to α:β T-cells and the second group with CD1d, which presents self-lipid antigens to CD1d-restricted natural killer T-cells (NKT) (84, 85). The fifth member CD1e shows characteristics from both groups (84). Most of the studies address CD1d-mediated antigen presentation in the liver. CD1d-restricted NKT cells are innate lymphocytes capable of immediate release of effector cytokines upon TCR stimuli. Type I NKT cells express a semi-invariant TCR and are therefore also called invariant NKT cells (iNKT), whereas type II NKT or diverse NKT cells express variable TCR (85). Several cell types in the liver are known to express CD1d molecules, present lipid moieties, and therefore activate iNKT cells. Cholangiocytes express CD1d in a consecutive manner and are able to activate iNKT cells *in vitro* (86). Moreover, HSCs pulsed with alpha-galactosylceramide (αGalCer), a model antigen for CD1d presentation, can also activate iNKT cells and result in IFNγ release (55). Additionally, HSCs can provide the activated NKT cells with IL-15 needed for further NKT cell proliferation (Figure 1B) (55). Hepatocytes also activate iNKT cells and can additionally control NKT cell survival (Figure 1A) (87). iNKT cells activated by αGalCer-pulsed hepatocytes release only IL-4, whereas activation of these cells by DCs leads additionally to the release of pro-inflammatory cytokine IFNγ (88). A release of IFNγ during hepatocyte presentation was only possible when exogenous IL-12 was added to the reaction (88). On the contrary, HBV-infected primary hepatocytes are able to activate NKT cells to produce IFNγ without any other stimulating cytokine (89).

Increased abundance or activation and cytokine release of NKT (innate lymphocyte) cell populations have been investigated in multiple liver diseases, and NKT cells have been considered as therapeutic targets (3, 90). On the other hand, liver biopsies from PSC, PBC, and alcoholic cirrhosis patients showed a decrease in CD1d expression compared to healthy livers (86). Another study reported that the loss of CD1d was more common in advanced PBC than in the early stage of PBC (91). Also CD1d expression on mouse hepatocytes is reduced in steatosis (87). These data might indicate antigen presentation changes in CD1d during liver diseases, but from the antigen presentation perspective the above-mentioned APCs have not been investigated in detail.

## Summary and Outlook

Taken together, in the liver microenvironment a complex network of various cell populations ensures the control of T-cell responses. Antigen presentation by non-professional APCs mostly results in T-cell tolerance or quiescent memory T-cell formation (Figure 1; Table 1). Additionally, an extensive regulatory apparatus in a bystander manner ensures the maintenance of the tolerogenic milieu in the liver (Figure 1). Importantly, these tolerogenic mechanisms do not hinder the ability of the liver to effectively respond to danger/infectious signals. While some of the non-professional APCs can trigger efficient T-cell responses, the generation of immunity is mostly related to DC populations in draining LN or monocyte-derived DCs in intrahepatic myeloid-cell aggregates for T-cell population expansion structures (92).

**Table 1:** Non-professional antigen-presenting cells and their immunoregulatory effect in the liver XP: cross-presentation; ?: not investigated in the liver; investigated in human (H) or in mouse (M) samples.

Cell type	Antigen presentation	Outcome	Immunoregulatory for bystander T cell activation	Reference
Liver sinusoidal endothelial cells (LSECs)	Major histocompatibility complex (MHC)-I, MHC-II, and XP	Induction of CD25^low^FoxP3^−^ regulatory T-cells (T_regs_) (M); LSEC primed CD8^+^ memory T-cells (M); and effector cytotoxic T-Lymphocyte (M)	Inhibition of T-cell activation (LSECtin) and dendritic cell (DC) antigen presentation (ICAM-1)	(27, 29, 30, 33, 36, 40)
Hepatocytes	MHC-I, CD1d, XP and MHC-II in inflammation	CD8^+^ T-cell Deletion (M); invariant natural killer cell (iNKT) activation (M); and T_reg_ induction (M)	?	(20, 45, 46, 50, 87)
Hepatic stellate cells	MHC-I, CD1d, MHC-II (in the presence of interferon γ)	T-cell response controversial (M) and iNKT activation (M)	CD4^+^ T_reg_ differentiation (retinoid acid) and inhibition of DC antigen presentation (CD54)	(54, 55, 63, 64)
Cholangiocytes	MHC-I, MHC class I-like-related molecule, and CD1d	CD8^+^ T-cell response controversial (M); mucosal-associated invariant T-cell activation (H); and iNKT activation *in vitro* (H + M)	?	(52, 68, 86)

Understanding the antigen presentation and consequent T-cell responses in the liver environment is especially important not only for the comprehension of liver diseases but also to uncover how the liver influences systemic immunity and tolerance.

## Author Contributions

CM performed literature search and wrote the manuscript and prepared the table and figure. SZ prepared the figure. MK supervised and critically read the manuscript. VL-K developed the concept of the manuscript, supervised, and wrote the manuscript.

## Conflict of Interest Statement

The authors declare that the research was conducted in the absence of any commercial or financial relationships that could be construed as a potential conflict of interest.

## Abbreviations

αGalCer, alpha-galactosylceramide; APC, antigen-presenting cell; CD, cluster of differentiation; CTL, cytotoxic T-Lymphocyte; DAMP, damage-associated molecular pattern; DC, dendritic cell; FoxP3, Forkhead-Box-Protein P3; GzmB, granzyme B; HBV, hepatitis B virus; HLA, human leukocyte antigen; HSC, hepatic stellate cell; IDO, indoleamine 2,3-dioxygenase; IL, interleukin; IFN, interferon; iMATE, intrahepatic myeloid-cell aggregates for T-cell population expansion; iNKT, invariant natural killer cell; KC, Kupffer cell; LEC, lymphatic endothelial cell; LRP-1, lipoprotein receptor-related protein 1; LSEC, liver sinusoidal endothelial cell; MAIT, mucosal-associated invariant T-cell; MHC, major histocompatibility complex; MR1, MHC class I-like-related molecule; OT-I/II cell, MHC class I/II-restricted ovalbumin-specific CD8^+^ T-cell; TGFβ, transforming growth factor beta; PBC, primary biliary cholangitis; PSC, primary sclerosing cholangitis; TCR, T-cell receptor; T_H_, helper T-cell; TLR, toll-like receptor; TNF, tumor necrosis factor; T_regs_, regulatory T-cell.

## References

[1] Crispe IN. Immune tolerance in liver disease. Hepatology (2014) 60(6):2109–17. 10.1002/hep.27254 24913836 PMC4274953

[2] Knolle PA Bottcher J Huang LR. The role of hepatic immune regulation in systemic immunity to viral infection. Med Microbiol Immunol (2015) 204(1):21–7. 10.1007/s00430-014-0371-0 25523194

[3] Doherty DG Immunity, tolerance and autoimmunity in the liver: a comprehensive review. J Autoimmun (2016) 66:60–75. 10.1016/j.jaut.2015.08.020 26358406

[4] Eipel C Abshagen K Vollmar B. Regulation of hepatic blood flow: the hepatic arterial buffer response revisited. World J Gastroenterol (2010) 16(48):6046–57. 10.3748/wjg.v16.i48.6046 21182219 PMC3012579

[5] Jenne CN Kubes P. Immune surveillance by the liver. Nat Immunol (2013) 14(10):996–1006. 10.1038/ni.2691 24048121

[6] Lalor PF Sun PJ Weston CJ Martin-Santos A Wakelam MJ Adams DH. Activation of vascular adhesion protein-1 on liver endothelium results in an NF-kappaB-dependent increase in lymphocyte adhesion. Hepatology (2007) 45(2):465–74. 10.1002/hep.21497 17256751

[7] Pinzani M Failli P Ruocco C Casini A Milani S Baldi E Fat-storing cells as liver-specific pericytes. Spatial dynamics of agonist-stimulated intracellular calcium transients. J Clin Invest (1992) 90(2):642–6. 10.1172/jci115905 1644929 PMC443145

[8] Blaner WS Hendriks HF Brouwer A de Leeuw AM Knook DL Goodman DS. Retinoids, retinoid-binding proteins, and retinyl palmitate hydrolase distributions in different types of rat liver cells. J Lipid Res (1985) 26(10):1241–51. 4067418

[9] Geerts A. History, heterogeneity, developmental biology, and functions of quiescent hepatic stellate cells. Semin Liver Dis (2001) 21(3):311–35. 10.1055/s-2001-17550 11586463

[10] Wang L Boyer JL The maintenance and generation of membrane polarity in hepatocytes. Hepatology (2004) 39(4):892–9. 10.1002/hep.20039 15057889

[11] Mönkemöller V Oie C Hubner W Huser T McCourt P. Multimodal super-resolution optical microscopy visualizes the close connection between membrane and the cytoskeleton in liver sinusoidal endothelial cell fenestrations. Sci Rep (2015) 5:16279. 10.1038/srep16279 26549018 PMC4637861

[12] Poisson J Lemoinne S Boulanger C Durand F Moreau R Valla D Liver sinusoidal endothelial cells: physiology and role in liver diseases. J Hepatol (2017) 66(1):212–27. 10.1016/j.jhep.2016.07.009 27423426

[13] Kempka G Kolb-Bachofen V. Binding, uptake, and transcytosis of ligands for mannose-specific receptors in rat liver: an electron microscopic study. Exp Cell Res (1988) 176(1):38–48. 10.1016/0014-4827(88)90118-8 3371424

[14] Ohtani Y Wang BJ Poonkhum R Ohtani O. Pathways for movement of fluid and cells from hepatic sinusoids to the portal lymphatic vessels and subcapsular region in rat livers. Arch Histol Cytol (2003) 66(3):239–52. 10.1679/aohc.66.239 14527165

[15] Ohtani O Ohtani Y Lymph circulation in the liver. Anat Rec (Hoboken) (2008) 291(6):643–52. 10.1002/ar.20681 18484610

[16] Lukacs-Kornek V. The role of lymphatic endothelial cells in liver injury and tumor development. Front Immunol (2016) 7:548. 10.3389/fimmu.2016.00548 27965673 PMC5127193

[17] Knolle PA. Staying local-antigen presentation in the liver. Curr Opin Immunol (2016) 40:36–42. 10.1016/j.coi.2016.02.009 26974478

[18] Grakoui A Crispe IN. Presentation of hepatocellular antigens. Cell Mol Immunol (2016) 13(3):293–300. 10.1038/cmi.2015.109 26924525 PMC4856799

[19] Magnusson S Berg T. Extremely rapid endocytosis mediated by the mannose receptor of sinusoidal endothelial rat liver cells. Biochem J (1989) 257(3):651–6. 10.1042/bj2570651 2930475 PMC1135637

[20] Ebrahimkhani MR Mohar I Crispe IN. Cross-presentation of antigen by diverse subsets of murine liver cells. Hepatology (2011) 54(4):1379–87. 10.1002/hep.24508 21721032 PMC3444257

[21] Hansen B Longati P Elvevold K Nedredal GI Schledzewski K Olsen R Stabilin-1 and stabilin-2 are both directed into the early endocytic pathway in hepatic sinusoidal endothelium via interactions with clathrin/AP-2, independent of ligand binding. Exp Cell Res (2005) 303(1):160–73. 10.1016/j.yexcr.2004.09.017 15572036

[22] Oie CI Appa RS Hilden I Petersen HH Gruhler A Smedsrod B Rat liver sinusoidal endothelial cells (LSECs) express functional low density lipoprotein receptor-related protein-1 (LRP-1). J Hepatol (2011) 55(6):1346–52. 10.1016/j.jhep.2011.03.013 21703209

[23] Ganesan LP Mates JM Cheplowitz AM Avila CL Zimmerer JM Yao Z Scavenger receptor B1, the HDL receptor, is expressed abundantly in liver sinusoidal endothelial cells. Sci Rep (2016) 6:20646. 10.1038/srep20646 26865459 PMC4749959

[24] Li R Oteiza A Sorensen KK McCourt P Olsen R Smedsrod B Role of liver sinusoidal endothelial cells and stabilins in elimination of oxidized low-density lipoproteins. Am J Physiol Gastrointest Liver Physiol (2011) 300(1):G71–81. 10.1152/ajpgi.00215.2010 21030611 PMC3025507

[25] Rubinstein D Roska AK Lipsky PE Liver sinusoidal lining cells express class II major histocompatibility antigens but are poor stimulators of fresh allogenic T lymphocytes. J Immunol (1986) 137:1803–10. 3489042

[26] Lohse AW Knolle PA Bilo K Uhrig A Waldmann C Ibe M Antigen-presenting function and B7 expression of murine sinusoidal endothelial cells and Kupffer cells. Gastroenterology (1996) 110:1175–81. 10.1053/gast.1996.v110.pm8613007 8613007

[27] Kruse N Neumann K Schrage A Derkow K Schott E Erben U Priming of CD4+ T cells by liver sinusoidal endothelial cells induces CD25low forkhead box protein 3-regulatory T cells suppressing autoimmune hepatitis. Hepatology (2009) 50(6):1904–13. 10.1002/hep.23191 19787806

[28] Carambia A Freund B Schwinge D Bruns OT Salmen SC Ittrich H Nanoparticle-based autoantigen delivery to Treg-inducing liver sinusoidal endothelial cells enables control of autoimmunity in mice. J Hepatol (2015) 62(6):1349–56. 10.1016/j.jhep.2015.01.006 25617499

[29] Limmer A Ohl J Kurts C Ljunggren H-G Reiss Y Groettrup M Efficient presentation of exogenous antigen by liver endothelial cells to CD8+ T-cells results in antigen specific T-cell tolerance. Nat Med (2000) 6(12):1348–54. 10.1038/82161 11100119

[30] Schurich A Bottcher JP Burgdorf S Penzler P Hegenbarth S Kern M Distinct kinetics and dynamics of cross-presentation in liver sinusoidal endothelial cells compared to dendritic cells. Hepatology (2009) 50(3):909–19. 10.1002/hep.23075 19610048

[31] Wohlleber D Kashkar H Gartner K Frings MK Odenthal M Hegenbarth S TNF-induced target cell killing by CTL activated through cross-presentation. Cell Rep (2012) 2(3):478–87. 10.1016/j.celrep.2012.08.001 22939982

[32] Berg M Wingender G Djandji D Hegenbarth S Momburg F Hammerling G Cross-presentation of antigens from apoptotic tumor cells by liver sinusoidal endothelial cells leads to tumor-specific CD8+ T cell tolerance. Eur J Immunol (2006) 36(11):2960–70. 10.1002/eji.200636033 17039564

[33] Diehl L Schurich A Grochtmann R Hegenbarth S Chen L Knolle PA. Tolerogenic maturation of liver sinusoidal endothelial cells promotes B7-homolog 1-dependent CD8+ T cell tolerance. Hepatology (2008) 47(1):296–305. 10.1002/hep.21965 17975811

[34] Böttcher JP Schanz O Garbers C Zaremba A Hegenbarth S Kurts C IL-6 trans-signaling-dependent rapid development of cytotoxic CD8+ T cell function. Cell Rep (2014) 8(5):1318–27. 10.1016/j.celrep.2014.07.008 25199826

[35] Wittlich M Dudek M Bottcher JP Schanz O Hegenbarth S Bopp T Liver sinusoidal endothelial cell cross-priming is supported by CD4 T cell-derived IL-2. J Hepatol (2017) 66(5):978–86. 10.1016/j.jhep.2016.12.015 28025060

[36] Böttcher JP Schanz O Wohlleber D Abdullah Z Debey-Pascher S Staratschek-Jox A Liver-primed memory T cells generated under noninflammatory conditions provide anti-infectious immunity. Cell Rep (2013) 3(3):779–95. 10.1016/j.celrep.2013.02.008 23499443

[37] Schurich A Berg M Stabenow D Bottcher J Kern M Schild HJ Dynamic regulation of CD8 T cell tolerance induction by liver sinusoidal endothelial cells. J Immunol (2010) 184(8):4107–14. 10.4049/jimmunol.0902580 20212092

[38] Ye B Liu X Li X Kong H Tian L Chen Y. T-cell exhaustion in chronic hepatitis B infection: current knowledge and clinical significance. Cell Death Dis (2015) 6:e1694. 10.1038/cddis.2015.42 25789969 PMC4385920

[39] Schölzel K Schildberg FA Welz M Borner C Geiger S Kurts C Transfer of MHC-class-I molecules among liver sinusoidal cells facilitates hepatic immune surveillance. J Hepatol (2014) 61(3):600–8. 10.1016/j.jhep.2014.04.028 24798625

[40] Tang L Yang J Liu W Tang X Chen J Zhao D Liver sinusoidal endothelial cell lectin, LSECtin, negatively regulates hepatic T-cell immune response. Gastroenterology (2009) 137(4):.e1–5. 10.1053/j.gastro.2009.07.051 19632227 PMC7127102

[41] Tang L Yang J Tang X Ying W Qian X He F. The DC-SIGN family member LSECtin is a novel ligand of CD44 on activated T cells. Eur J Immunol (2010) 40(4):1185–91. 10.1002/eji.200939936 20127679

[42] Uhrig A Banafsche R Kremer M Hegenbarth S Hamann A Neurath M Development and functional consequences of LPS tolerance in sinusoidal endothelial cells of the liver. J Leukoc Biol (2005) 77(5):626–33. 10.1189/jlb.0604332 15860798

[43] Warren A Le Couteur DG Fraser R Bowen DG McCaughan GW Bertolino P. T lymphocytes interact with hepatocytes through fenestrations in murine liver sinusoidal endothelial cells. Hepatology (2006) 44(5):1182–90. 10.1002/hep.21378 17058232

[44] Warren A Bertolino P Benseler V Fraser R McCaughan GW Le Couteur DG. Marked changes of the hepatic sinusoid in a transgenic mouse model of acute immune-mediated hepatitis. J Hepatol (2007) 46(2):239–46. 10.1016/j.jhep.2006.08.022 17125874

[45] Bertolino P Trescol-Biémont M-C Rabourdin-Combe C. Hepatocytes induce functional activation of naive CD8+ T lymphocytes but fail to promote survival. Eur J Immunol (1998) 28:221–36. 10.1002/(SICI)1521-4141(199801)28:01<221::AID-IMMU221>3.0.CO;2-F 9485202

[46] Holz LE Benseler V Bowen DG Bouillet P Strasser A O’Reilly L Intrahepatic murine CD8 T-cell activation associates with a distinct phenotype leading to Bim-dependent death. Gastroenterology (2008) 135(3):989–97. 10.1053/j.gastro.2008.05.078 18619445 PMC2956118

[47] Dolina JS Cechova S Rudy CK Sung SJ Tang WW Lee J Cross-presentation of soluble and cell-associated antigen by murine hepatocytes is enhanced by collectrin expression. J Immunol (2017) 198(6):2341–51. 10.4049/jimmunol.1502234 28159899

[48] Ochel A Cebula M Riehn M Hillebrand U Lipps C Schirmbeck R Effective intrahepatic CD8+ T-cell immune responses are induced by low but not high numbers of antigen-expressing hepatocytes. Cell Mol Immunol (2016) 13(6):805–15. 10.1038/cmi.2015.80 26412123 PMC5101449

[49] Tay SS Wong YC McDonald DM Wood NAW Roedinger B Sierro F Antigen expression level threshold tunes the fate of CD8 T cells during primary hepatic immune responses. Proc Natl Acad Sci U S A (2014) 111(25):E2540–9. 10.1073/pnas.1406674111 24927525 PMC4078818

[50] Herkel J Jagemann B Wiegard C Lazaro JF Lueth S Kanzler S MHC class II-expressing hepatocytes function as antigen-presenting cells and activate specific CD4 T lymphocytes. Hepatology (2003) 37(5):1079–85. 10.1053/jhep.2003.50191 12717388

[51] Luth S Huber S Schramm C Buch T Zander S Stadelmann C Ectopic expression of neural autoantigen in mouse liver suppresses experimental autoimmune neuroinflammation by inducing antigen-specific Tregs. J Clin Invest (2008) 118(10):3403–10. 10.1172/JCI32132 18802476 PMC2542846

[52] Derkow K Loddenkemper C Mintern J Kruse N Klugewitz K Berg T Differential priming of CD8 and CD4 T-cells in animal models of autoimmune hepatitis and cholangitis. Hepatology (2007) 46(4):1155–65. 10.1002/hep.21796 17657820

[53] Derkow K Müller A Eickmeier I Seidel D Rust Moreira MV Kruse N Failure of CD4 T-cells to respond to liver-derived antigen and to provide help to CD8 T-cells. PLoS One (2011) 6(7):e21847. 10.1371/journal.pone.0021847 21779338 PMC3136477

[54] Vinas O Bataller R Sancho-Bru P Ginès P Berenguer C Enrich C Human hepatic stellate cells show features of antigen-presenting cells and stimulate lymphocyte proliferation. Hepatology (2003) 38:919–29. 10.1002/hep.1840380418 14512879

[55] Winau F Hegasy G Weiskirchen R Weber S Cassan C Sieling PA Ito cells are liver-resident antigen-presenting cells for activating T cell responses. Immunity (2007) 26(1):117–29. 10.1016/j.immuni.2006.11.011 17239632

[56] Jiang G Yang HR Wang L Wildey GM Fung J Qian S Hepatic stellate cells preferentially expand allogeneic CD4+ CD25+ FoxP3+ regulatory T cells in an IL-2-dependent manner. Transplantation (2008) 86(11):1492–502. 10.1097/TP.0b013e31818bfd13 19077880 PMC2888269

[57] Bomble M Tacke F Rink L Kovalenko E Weiskirchen R. Analysis of antigen-presenting functionality of cultured rat hepatic stellate cells and transdifferentiated myofibroblasts. Biochem Biophys Res Commun (2010) 396(2):342–7. 10.1016/j.bbrc.2010.04.094 20403338

[58] Yu MC Chen CH Liang X Wang L Gandhi CR Fung JJ Inhibition of T-cell responses by hepatic stellate cells via B7-H1-mediated T-cell apoptosis in mice. Hepatology (2004) 40(6):1312–21. 10.1002/hep.20488 15565659

[59] Chinnadurai R Grakoui A. B7-H4 mediates inhibition of T cell responses by activated murine hepatic stellate cells. Hepatology (2010) 52(6):2177–85. 10.1002/hep.23953 21064155 PMC2995273

[60] Charles R Chou HS Wang L Fung JJ Lu L Qian S. Human hepatic stellate cells inhibit T-cell response through B7-H1 pathway. Transplantation (2013) 96(1):17–24. 10.1097/TP.0b013e318294caae 23756770 PMC3696433

[61] Sumpter TL Dangi A Matta BM Huang C Stolz DB Vodovotz Y Hepatic stellate cells undermine the allostimulatory function of liver myeloid dendritic cells via STAT3-dependent induction of IDO. J Immunol (2012) 189(8):3848–58. 10.4049/jimmunol.1200819 22962681 PMC3466356

[62] Höchst B Schildberg FA Sauerborn P Gabel YA Gevensleben H Goltz D Activated human hepatic stellate cells induce myeloid derived suppressor cells from peripheral blood monocytes in a CD44-dependent fashion. J Hepatol (2013) 59(3):528–35. 10.1016/j.jhep.2013.04.033 23665041

[63] Dunham RM Thapa M Velazquez VM Elrod EJ Denning TL Pulendran B Hepatic stellate cells preferentially induce Foxp3+ regulatory T cells by production of retinoic acid. J Immunol (2013) 190(5):2009–16. 10.4049/jimmunol.1201937 23359509 PMC3575565

[64] Schildberg FA Wojtalla A Siegmund SV Endl E Diehl L Abdullah Z Murine hepatic stellate cells veto CD8 T cell activation by a CD54-dependent mechanism. Hepatology (2011) 54(1):262–72. 10.1002/hep.24352 21488077

[65] Leon MP Kirby JA Gibbs P Burt AD Bassendine MF. Immunogenicity of biliary epithelial cells: study of the expression of B7 molecules. J Hepatol (1995) 22:591–5. 10.1016/0168-8278(95)80456-0 7544369

[66] Leon MP Bassendine MF Wilson JL Simi A Thick M Kirby JA Immunogenecity of biliary epithelium: investigation of antigen presentation to CD4+ T cells. Hepatology (1996) 24:561–7. 10.1002/hep.510240317 8781325

[67] Ayres RC Neuberger JM Shaw J Joplin R Adams DH. Intercellular adhesion molecule-1 and MHC antigens on human intrahepatic bile duct cells: effect of pro-inflammatory cytokines. Gut (1993) 34(9):1245–9. 10.1136/gut.34.9.1245 8104850 PMC1375463

[68] Jeffery HC van Wilgenburg B Kurioka A Parekh K Stirling K Roberts S Biliary epithelium and liver B cells exposed to bacteria activate intrahepatic MAIT cells through MR1. J Hepatol (2016) 64(5):1118–27. 10.1016/j.jhep.2015.12.017 26743076 PMC4822535

[69] Le Bourhis L Martin E Peguillet I Guihot A Froux N Core M Antimicrobial activity of mucosal-associated invariant T cells. Nat Immunol (2010) 11(8):701–8. 10.1038/ni.1890 20581831

[70] Kjer-Nielsen L Patel O Corbett AJ Le Nours J Meehan B Liu L MR1 presents microbial vitamin B metabolites to MAIT cells. Nature (2012) 491(7426):717–23. 10.1038/nature11605 23051753

[71] Kurioka A Walker LJ Klenerman P Willberg CB. MAIT cells: new guardians of the liver. Clin Transl Immunol (2016) 5(8):e98. 10.1038/cti.2016.51 27588203 PMC5007630

[72] Lukacs-Kornek V Malhotra D Fletcher AL Acton SE Elpek KG Tayalia P Regulated release of nitric oxide by nonhematopoietic stroma controls expansion of the activated T cell pool in lymph nodes. Nat Immunol (2011) 12(11):1096–104. 10.1038/ni.2112 21926986 PMC3457791

[73] Hirosue S Vokali E Raghavan VR Rincon-Restrepo M Lund AW Corthesy-Henrioud P Steady-state antigen scavenging, cross-presentation, and CD8+ T cell priming: a new role for lymphatic endothelial cells. J Immunol (2014) 192(11):5002–11. 10.4049/jimmunol.1302492 24795456 PMC4025611

[74] Cohen JN Guidi CJ Tewalt EF Qiao H Rouhani SJ Ruddell A Lymph node-resident lymphatic endothelial cells mediate peripheral tolerance via Aire-independent direct antigen presentation. J Exp Med (2010) 207(4):681–8. 10.1084/jem.20092465 20308365 PMC2856027

[75] Fletcher AL Lukacs-Kornek V Reynoso ED Pinner SE Bellemare-Pelletier A Curry MS Lymph node fibroblastic reticular cells directly present peripheral tissue antigen under steady-state and inflammatory conditions. J Exp Med (2010) 207(4):689–97. 10.1084/jem.20092642 20308362 PMC2856033

[76] Podgrabinska S Kamalu O Mayer L Shimaoka M Snoeck H Randolph GJ Inflamed lymphatic endothelium suppresses dendritic cell maturation and function via Mac-1/ICAM-1-dependent mechanism. J Immunol (2009) 183(3):1767–79. 10.4049/jimmunol.0802167 19587009 PMC4410990

[77] Farrell DJ Hines JE Walls AF Kelly PJ Bennett MK Burt AD. Intrahepatic mast cells in chronic liver diseases. Hepatology (1995) 22(4 Pt 1):1175–81. 10.1016/0270-9139(95)90627-4 7557869

[78] Lotfi-Emran S Ward BR Le QT Pozez AL Manjili MH Woodfolk JA Human mast cells present antigen to autologous CD4(+) T cells. J Allergy Clin Immunol (2018) 141:311–21. 10.1016/j.jaci.2017.02.048 28624612

[79] Jones H Hargrove L Kennedy L Meng F Graf-Eaton A Owens J Inhibition of mast cell-secreted histamine decreases biliary proliferation and fibrosis in primary sclerosing cholangitis MDR2-/- mice. Hepatology (2016) 64(4):1202–16. 10.1002/hep.28704/suppinfo 27351144 PMC5033697

[80] Hargrove L Kennedy L Demieville J Jones H Meng F DeMorrow S Bile duct ligation-induced biliary hyperplasia, hepatic injury, and fibrosis are reduced in mast cell-deficient kit(W-sh) mice. Hepatology (2017) 65(6):1991–2004. 10.1002/hep.29079/suppinfo 28120369 PMC5444972

[81] Geng S Matsushima H Okamoto T Yao Y Lu RS Page K Emergence, origin, and function of neutrophil-dendritic cell hybrids in experimentally induced inflammatory lesions in mice. Blood (2013) 121(10):1690–700. 10.1182/blood-2012-07445197 23305733 PMC3591794

[82] Vono M Lin A Norrby-Teglund A Koup RA Liang F Loré K. Neutrophils acquire the capacity for antigen presentation to memory CD4+ T cells in vitro and ex vivo. Blood (2017) 129(14):1991–2001. 10.1182/blood-2016-10744441 28143882 PMC5383872

[83] Brigl M Brenner MB. CD1: antigen presentation and T cell function. Annu Rev Immunol (2004) 22:817–90. 10.1146/annurev.immunol.22.012703.104608 15032598

[84] De Libero G Mori L. Novel insights into lipid antigen presentation. Trends Immunol (2012) 33(3):103–11. 10.1016/j.it.2012.01.005 22342205

[85] Mori L Lepore M De Libero G. The immunology of CD1- and MR1-restricted T cells. Annu Rev Immunol (2016) 34:479–510. 10.1146/annurev-immunol-032414-112008 26927205

[86] Schrumpf E Tan C Karlsen TH Sponheim J Björkström NK Sundens O The biliary epithelium presents antigens to and activates natural killer T cells. Hepatology (2015) 62:1249–59. 10.1002/hep.27840/suppinfo 25855031 PMC4589438

[87] Zeissig S Peuker K Iyer S Gensollen T Dougan SK Olszak T CD1d-restricted pathways in hepatocytes control local natural killer T cell homeostasis and hepatic inflammation. Proc Natl Acad Sci U S A (2017) 114(39):10449–54. 10.1073/pnas.1701428114 28893990 PMC5625893

[88] Trobonjaca Z Leithauser F Moller P Schirmbeck R Reimann J Activating immunity in the liver. I. liver dendritic cells (but not hepatocytes) are potent activators of IFN-γ release by liver NKT cells. J Immunol (2001) 167(3):1413–22. 10.4049/jimmunol.167.3.1413 11466360

[89] Zeissig S Murata K Sweet L Publicover J Hu Z Kaser A Hepatitis B virus-induced lipid alterations contribute to natural killer T cell-dependent protective immunity. Nat Med (2012) 18(7):1060–8. 10.1038/nm.2811 22706385 PMC3478098

[90] Hung JT Huang JR Yu AL. Tailored design of NKT-stimulatory glycolipids for polarization of immune responses. J Biomed Sci (2017) 24(1):22. 10.1186/s12929-017-0325-0 28335781 PMC5364570

[91] Tsuneyama K Yasoshima M Harada K Hiramatsu K Gershwin ME Nakanuma Y. Increased CD1d expression on small bile duct epithelium and epithelioid granuloma in livers in primary biliary cirrhosis. Hepatology (1998) 28:620–3. 10.1002/hep.510280303 9731549

[92] Huang LR Wohlleber D Reisinger F Jenne CN Cheng RL Abdullah Z Intrahepatic myeloid-cell aggregates enable local proliferation of CD8(+) T cells and successful immunotherapy against chronic viral liver infection. Nat Immunol (2013) 14(6):574–83. 10.1038/ni.2573 23584070

